# Gut micobiota alteration by *Lactobacillus rhamnosus* reduces pro-inflammatory cytokines and glucose level in the adult model of Zebrafish

**DOI:** 10.1186/s13104-021-05706-5

**Published:** 2021-08-09

**Authors:** Fatemeh Bootorabi, Farshid Saadat, Reza Falak, Hamed Manouchehri, Reza Changizi, Hasan Mohammadi, Farnaz Safavifar, Mohammad Reza Khorramizadeh

**Affiliations:** 1 Biosensor Research Center, Endocrinology and Metabolism Molecular-Cellular Sciences Institute, Tehran University of Medical Sciences, Endocrinology and Metabolism Research Institute (EMRI), Next to Dr. Shariati Hospital,#10 Jalal Al-E-Ahmad Expy, 1411713119 Tehran, Iran; 2 Department of Immunology, Faculty of Medicine, Guilan University of Medical Sciences, Rasht, Iran; 3 Department of Immunology, School of Medicine, Iran University of Medical Sciences, Tehran, Iran; 4 Department of Aquaculture, Babol Branch of Islamic Azad University, 47134 Babol, Iran; 5 Zebrafish Core Facility, Endocrinology and Metabolism Research Institute (EMRI), Tehran University of Medical Sciences, Tehran, Iran; 6 Endocrinology and Metabolism Research Center, Endocrinology and Metabolism Clinical Sciences Institute, Tehran University of Medical Sciences, Tehran, Iran

**Keywords:** Cytokine, *Lactobacillus rhamnosus*, Probiotic, Type 2 diabetes mellitus (T2DM), Zebrafish

## Abstract

**Objective:**

Type 2 diabetes mellitus (T2DM) is still a challenge for physicians to manage patient’s circumstances. It is assumed that alterations in the normal flora may be involved in the pathogenesis of T2DM through inducing chronic inflammation. To investigate the effect of *Lactobacillus rhamnosus* as a common probiotic on T2DM, we induced an experimental model of T2DM in adult male Zebrafish by gradient hyper-glucose accumulation methodology.

**Results:**

In this trial 3-month old male adult Zebrafish were divided in to four groups including two control groups and T2DM induced groups with or without probiotic treatment. After 5 days of acclimation, T2DM was induced by a gradient hyper-glucose accumulation methodology. Diabetic fishes had statistically abnormal blood glucose and pro-inflammatory cytokine levels compared to control group (p = 0.0001). These results suggest that probiotic intervention decreased the blood glucose level in the T2DM-P group by decreasing pro-inflammatory cytokines responsible for signaling in T2DM therapeutic modalities.

**Supplementary Information:**

The online version contains supplementary material available at 10.1186/s13104-021-05706-5.

## Introduction

Metabolic syndrome has steadily increased globally and many etiological factors including altered inflammatory states, adipose abnormalities and insulin resistance, contribute to their pathogenesis and thus should be considered in related basic studies [1–3]. Diabetes mellitus (DM) which is known by glycemic disturbances is usually categorized to various categories. Type 1 DM patients are characterized by destruction of insulin-producing pancreatic beta-cells and type 2 diabetes mellitus (T2DM) patients with lowered response to insulin. In T2DM, an individual’s lifestyle and genetic coding are key risk factors [4, 5]. In obese T2DM patients, it was shown that losing weight reduces the glucose levels and can also improve glycemic control [6, 7]. However, there are still multiple challenges for physicians to manage patients’ condition, including side effects and toxicity of current therapeutics and medication failures [8]. Recently, several studies have found that changes in gut microbiota composition play a role in the development of T2DM and obesity [1, 9]. Furthermore, nowadays it is useful to realize the effects of probiotics on diabetes management.

Probiotics are defined as live microorganisms with healthy beneficial to host if up taken in adequate amounts [10, 11]. Recent advances have highlighted the beneficial outcome in the pathology of inflammatory disorders after using gastrointestinal bacteria therapeutically [12]. Much evidence suggests that probiotic uptake has beneficial effects on blood glucose through different mechanisms [13–16]. Since intestinal microbiota and their metabolites directly contribute in insulin resistance; probiotic could have a strong effect on physiological function by altering gastrointestinal bacterial community [17, 18]. Consumption of a sufficient amount of probiotic is considered a therapeutic method for weight reduction and control of T2DM [19]. Furthermore, there is a direct correlation between the anti-diabetic drugs and gut microbiome community [9].

T2DM and obesity in the context of epidemiological data provide a solid platform for in vivo investigation of the disease. Nowadays, zebrafish (*Danio rerio*) has emerged as a powerful tool for scientific community among vertebrates. In the beginning of embryogenesis, zebrafish has transparent embryos with more than 85% of genetic similarity with human activity and thus as an attractive model organism for biomedical research [20]. Besides, it is also widely used for investigations on probiotics and host interaction with different gut microbiota and infectious diseases [21, 22].

Here in, we have developed a diet-induced zebrafish model for diabetes, to investigate the potential effect of oral administration of probiotic *Lactobacillus rhamnosus* as a diet supplement for inflammation caused by T2DM. If probiotic intervention could hinder the blood glucose elevation in the T2DM model via impact on cytokine levels, it might be considered a novel therapeutic approach in T2DM treatment.

## Main text

### Materials and methods

#### Animals

Three months old adult male fish as a gift from Zebrafish core facility, University of Tampere, Finland were divided into four groups including healthy control (HC), healthy control received probiotic (HC-P), T2DM group (T2DM) and T2DM group received probiotic (T2DM-P). Fish acclimation, induction diabetes and biometric analysis are described in Additional file 1 [23, 24].

#### Probiotic administration

Administration of *purchased L. rhamnosus* GG (ATCC: 53103) capsule from Culturelle Probiotics Co., Canada; is described in Additional file 1.

#### Blood glucose measurements

The blood collection used for glucometery using Match™ (OK Biotech Co, Taiwan) is described in Additional file 1.

#### Histological staining

After collecting biometric results, the small intestine of same fish used for histological staining procedures [25, 26] which is described in Additional file 1.

#### Quantitative real-time polymerase chain reaction analysis

Relative expression of IL-1β and TNF-α (primer sequences in Additional file 2: Table S1) levels were calculated using ΔΔCt method [27] using reverse transcription technique which is described in Additional file 1.

#### Statistical analysis

Data are presented as mean ± standard error. Student’s t-test was used for comparison between the two experimental groups using the statistical software package SPSS Statistics version 16 (IBM Corp., Armonk, NY, USA) with significance accepted at p < 0.001.

### Results

#### Zebrafish biometric analysis

As summarized in Additional file 3: Table S2, a significant decrease in the length of the diabetic group (T2DM) and probiotic-treated diabetics (T2DM-P) was observed compared to controls (HC). Moreover, we observed a slight increase in the length of the probiotic-treated controls (HC-P vs. HC, P = 0.013). Although both diabetic groups (T2DM and T2DM-P) showed lower weight compared to controls; the statistical analysis showed a significant difference between T2DM and HC (P = 0.0001). As shown in Additional file 3: Table S2, a significant increase in the BMI of the HC-P group (1.255-fold) and a significant decrease in the T2DM group (0.343-fold) compared with HC (p < 0.05) was observed.

#### Impact of probiotics on blood glucose

**Fig. 1:**
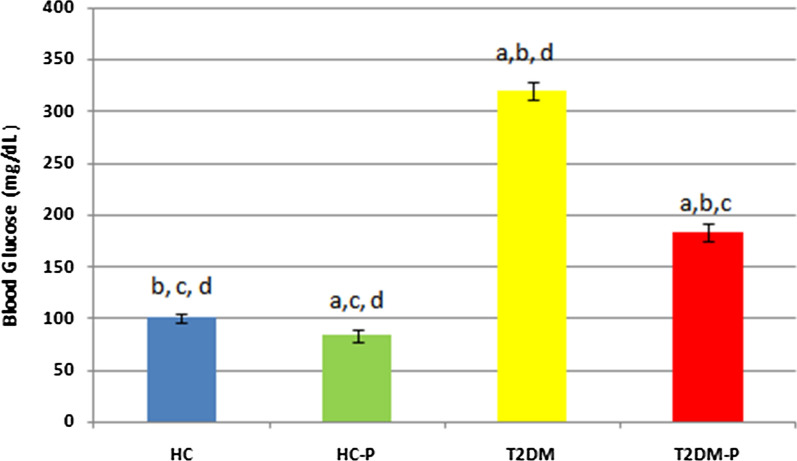
This diagram represents the result of blood glucose from all trail groups. Data represents the mean + SE of the results for three independent assays. Statistical analysis compares difference of the mean of results between T2DM-P and HC, HC-P and T2DM. **a** Significant difference of other groups vs. HC. **b** Significant difference of other groups vs. HC-P. **c** Significant difference of other groups vs. T2DM. **d** Significant difference of other groups vs. T2DM-P Difference with p < 0.001 was considered significant. Each group contained 6 zebrafish

We observed significant blood glucose elevation in the T2DM group compared with HC and HC-P groups (P < 0.0001, Fig. 1). After 10 days treatment with probiotic, blood glucose has been decreased in the HC-P group in correlation with the supplemented probiotic (P = 0.011).

#### Influence of probiotics on zebrafish intestine

**Fig. 2:**
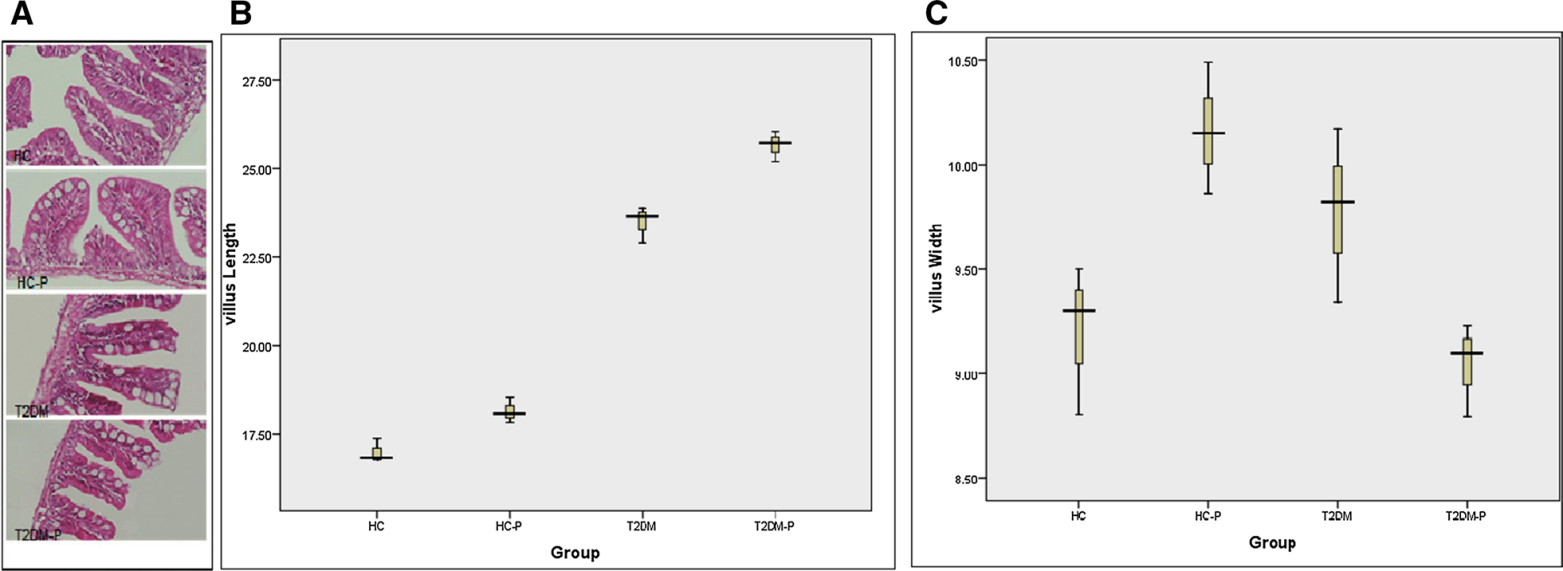
**A** Histopathology evaluation of zebrafish small intestine. Intestinal tissues were stained with H&E and studied by microscopy (× 400 resolution). Healthy control group (HC), Healthy control group supplemented with probiotics (HC-P), Diabetic group (T2DM), Diabetic group supplemented with probiotic (T2DM-P). **B** Villus length increased slightly in the T2DM-P group following probiotic supplementation compared to T2DM group. **C**. Villus width was slightly higher in the T2DM group compared to T2DM-P group. Data shows mean ± SE for three independent assays

Histological analysis revealed some visible changes in the villus width and length during the probiotic treatment. Villus length has increased slightly in the T2DM-P group compared to T2DM; conversely, villus width has slightly increased in the T2DM group compared with the T2DM-P (Fig. 2). Histological analysis of the intestinal tissues indicated a wide hyperplasia in the goblet cells located in microvilli’s of the T2DM and T2DM-P groups.

#### Influence of probiotics on zebrafish pro-inflammatory cytokine expression

**Fig. 3:**
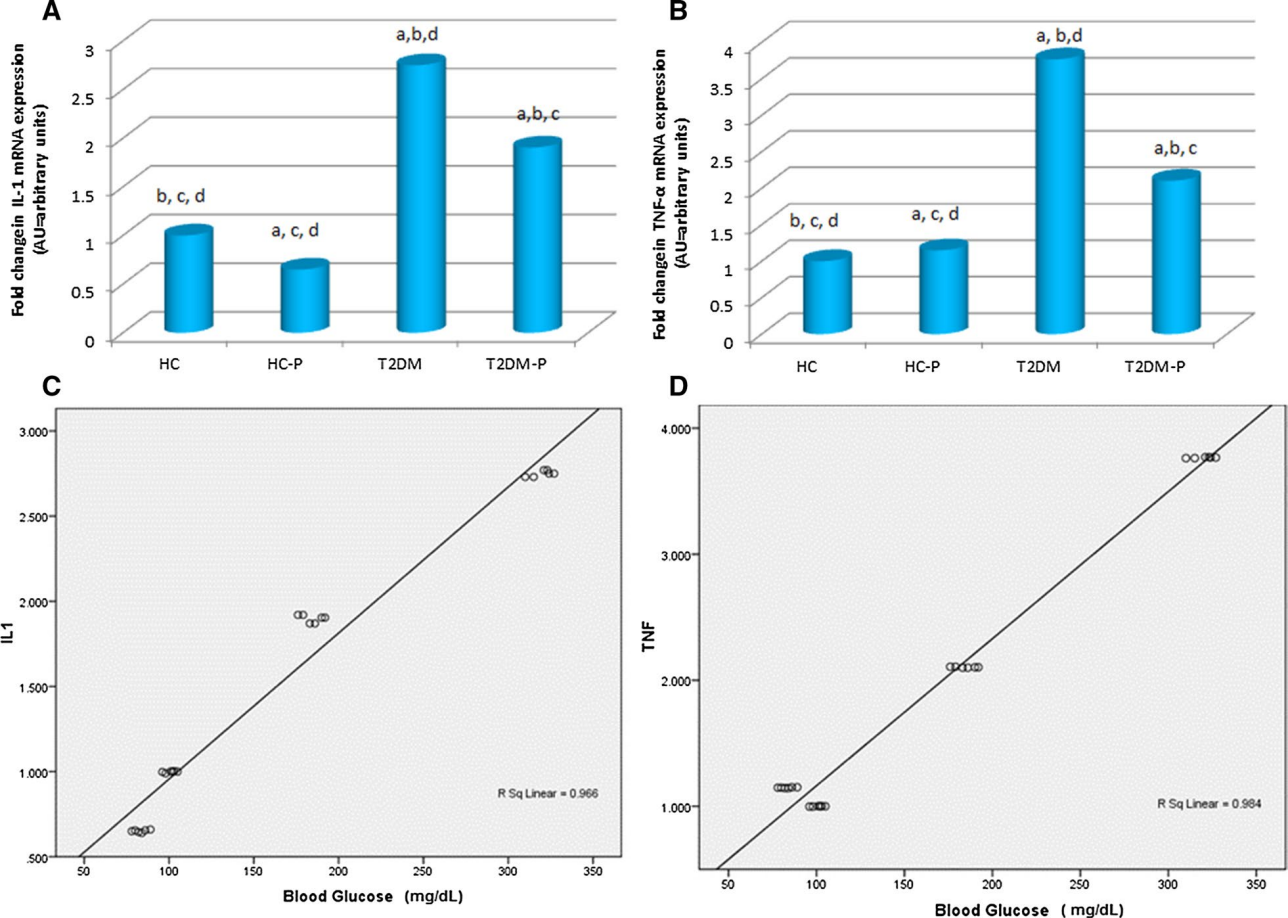
The relative expression of the intestinal pro-inflammatory cytokines after 10 days probiotic intervention. **A** The effects of probiotic on mRNA expression of IL-1β were determined by PCR. **B** The relative expression of TNF-α from all trail groups represent. The expression levels of β-actin were used as a loading control. **C** The Correlation of IL-1β to blood glucose. **D** The Correlation of TNF-α vs. blood glucose. Control group (HC), Control + Probiotic (HC-P), Diabetic group (T2DM), Diabetic group with probiotic(T2DM-P) groups. a Significant difference of other groups vs. HC. b Significant difference of other groups vs. HC-P. c Significant difference of other groups vs. T2DM. d Significant difference of other groups vs. T2DM-P The mean difference is significant at p < 0.001

The relative expression of pro-inflammatory cytokines, IL-1β and TNF-α was depicted in Fig. 3A, B. Compared to the control group (HC and HC-P, Fig. 3A), both cytokines were over-expressed in the T2DM group (P < 0.0001). The expression levels of IL-1β and TNF-α increased by 2.75 and 3.77-fold, respectively, in the T2DM group compared with HC. We found that probiotic supplementation resulted in a significant decrease in the expression levels of pro-inflammatory cytokines in T2DM-P group compare to T2DM (P < 0.001).

#### Correlation between blood glucose and pro-inflammatory cytokine

The correlation between blood glucose in various groups of zebrafish and pro-inflammatory cytokines (IL-1β and TNF-α) expression was r =  − 0.966, p = 0.001 and r =  − 0.984, p = 0.001, respectively (Fig. 3C, D).

### Discussion

A variety of genetic and feeding models of diabetes have been established, nevertheless, the majority of them have shown an inconsistency in pathological defects compared to human disease. Therefore, among creatures, zebrafish gained a growing platform for developmental research on diseases modeling, based on high similarity of digestive tract and comparable microbial and gut colonization with human [28–30]. Although, adult zebrafish has not been generally applied as an experimental model to study diabetes; here, we have well established it by overfeeding at adult stage, to study the effects of probiotic supplements on T2DM in vivo model.

To generate this model, we used a gradient hyper-glucose accumulation methodology. Physiologically, adult zebrafish absorb molecules from water to make a hyperosmotical internal environment, therefore, immersing them in a glucose containing solution (starting from 50 mM) and rising to final 200 mM could be beneficial to obtain a model of hyperglycemia in a much shorter time than other stepwise elevating glucose concentration of T2DM model [31]. According to our findings, this protocol increases blood glucose up to 300 mg/dL in T2DM Zebrafish which is in accordance to Gleeson et al. who showed a up to 400 mg/dL increase in blood glucose of adult zebrafish immersed in a 1% glucose solution [32]. Moreover, this method provides micro-environment stability and prevents fish fatality.

In the biometric result, probiotic-supplemented diet improved fish weight compared with the standard diet in both T2DM and HC groups similar to observations of Valcarce et al. [30]. Although, we did not expect to see significant changes on the length of the fish during the treatment periods, our data indicated a slight increase in the length and calculated body mass index (BMI) of HC-P and T2DM-P groups. Typically BMI as an excess of body fat is a marker of obesity and obesity related diseases. Although, the pathogenesis of obesity and its association to metabolic disorders like T2DM is complicated, adipose tissue polarized macrophages to increase secretion of pro‐inflammatory cytokines such as TNF-α. Moreover, gut microbiota alterations through probiotic is considered to induce the host inflammatory response. As we shown in Fig. 3B, TNF-α was increased 1.149 in healthy group treated by probiotic. It is assumed that such a small increment of cytokine expression is not able to completely activate the entire inflammatory pathway like chronic inflammation process induced by obesity [33]. Falcinelli et al. exposed adult zebrafish to different amounts of lipid to understand the correlation between microbiota and obesity. They indicated that high lipid contents reduced community diversity which affected the genes transcription which involved in appetite control and supplementation of the diet with Lactobacillus rhamnosus decreases total body cholesterol in zebrafish [34]. In addition to TNF-α, IL-1 is another pro-inflammatory cytokines which released in obese animal via M1 macrophage stimulation [35]. According to our data, a significant decrement of IL-1 was also occurred in HC-P group compared to HC (1.533) which might be explained the probiotic modality on whole cytokine profile alteration in experimental model. This finding remarkably points out to the strong value of probiotic and contributes with the physiological condition which enhances effective fish development and growth [14, 19].

Our histological analysis clearly showed some visible changes in the villus length and width during the probiotic treatment. Since the T2DM fish groups were under harsh condition due to high concentration of glucose, their homeostasis tries to adapt the condition to survive by extension of the villus diameter to increase the absorption. Additionally, result of intestinal staining specified hyperplasia in goblet cells located in microvillis’ in the T2DM group. As, one of the primary characteristic sign of obesity is hyperplasia and hypertrophy; administration of probiotic had such an ability to prevent goblet cells disruption due to high glucose in digestion system of our zebrafish diabetic model [36, 37].

Alterations in the intestinal homoeostasis may play a major role in the development of systemic inflammatory diseases including diabetes in which blood glucose management is challenging [38, 39]. In parallel with previous research, we showed that the consumption of probiotic based foods had significantly decreased blood glucose [40, 41]. Moreover, we identified the blood glucose level slightly lower in the HC-P group than the others. Since probiotic can strongly affect the growth, development and immune system improvement; thus, current results specified the probiotic *Lactobacillus rhamnosus* capability to improve tolerance in high glucose concentration [40].

Recently, the contribution of the mucosal immune system and the gut microbiome in metabolic disease including T2DM has been highly concerned [38, 42]. Our findings showed that relative mRNA expression levels of IL1-β and TNF-α were down-regulated in fish with probiotic supplementation despite the induction of innate immune-related cytokine genes by probiotic *Chromobacterium aquaticum* reported by Yi et al. [43]. This apparent inconsistency in cytokine profile was seen among the genus of *Lactobacillus* whereas *L. sakei* induced pro-inflammatory cytokines including IL-1β and TNF-α; and *L. johnsonii* promoted the production of TGF-β in cellular models [44].

Moreover, there was a robust positive correlation between both IL1-β and TNF-α and blood glucose levels (Fig. 3C, D). Delgadillo-Silva et al. showed that altered composition of the gut microbiome stimulate the intestinal residing innate and adaptive immune cells and induce a cytokine-mediated inflammation which is accompanied by hyperglycemia [45]. TNF as an inhibitor of insulin signaling is a major contributor towards obesity-related diseases. Indeed, it has been demonstrated that mentioned cytokines may play role in the inflammatory destruction of insulin-producing β-cells in human T2DM [39, 46, 47]. Due to their synergistic effect, pharmacological blockage of mentioned cytokines has been clinically modulating inflammatory diseases; however, a therapeutic gap for managing islet inflammation and cytokine production in T2DM is present [48].

Finally, we assumed that probiotic bacteria, like *L. rhamnosus*, through their pathogen associated molecular pattern signaling pathway and bioactive components might reduce the immune-cell infiltration, decrease pro-inflammatory cytokines and ameliorate the hyperglycemic phenotype n fish models. Hence, probiotic intervention might be useful in blood glucose management in T2DM patients.

### Conclusion

In summary, this study elucidates that probiotics such as *Lactobacillus rhamnosus* may hinder the blood glucose elevation in the T2DM group by their immunemodulatory effects. Thereby development of a probiotic based therapeutic formulation could promote healthiness in patients with T2DM.

### Limitations

The limitation of this study includes lack of data on time dependent changes in blood glucose levels, cytokines levels and comparing different type of probiotics. Therefore, the effectiveness of various probiotic and their long-term impact on glucose levels should be investigated in this useful experimental model.

## Supplementary Information

**Additional file 1**. Complementary information of animals, probiotic administration, histological staining and quantitative real-time polymerase chain reaction analysis.

**Additional file 2: Table S1**. Primer sequences used for amplification of specific genes through real-time quantitative PCR.

**Additional file 3**: **Table S2**. Summary of the effects of probiotic supplementation on length, weight gain and BMI of experimental groups.

## Data Availability

The data supporting our findings be presented within the manuscript and additional supporting files. Moreover, other datasets used during the current study are available from the corresponding author on reasonable request.

## Abbreviations

ATCC: American Type Culture Collection
BMI: Body mass index
BP: Base pair
CFU: Colony forming unit
EMRI: Endocrinology and Metabolism Research Institute
HC: Healthy control group received no probiotic treatment
HC-P: Healthy control group treated with probiotic
H&E: Hematoxylin and eosin
IL-1β: Interleukin 1 *beta*
Real-time PCR: Real-time polymerase chain reaction
SE: Standard error
T2DM: Type 2 diabetes mellitus
T2DM: T2DM group received no probiotic supplement
T2DM-P: T2DM group received probiotic supplements
TGF-β: Transforming growth factor beta
TNF-α: Tumor necrosis factor alpha

## References

[1] Komaroff AL (2017). The microbiome and risk for obesity and diabetes. JAMA.

[2] Gong M, Wen S, Nguyen T (2020). Converging relationships of obesity and hyperuricemia with special reference to metabolic disorders and plausible therapeutic implications. Diabetes Metab Syndrome Obesity Targets Ther.

[3] Manne ND, Ginjupalli GK, Rice KM (2020). Long-term treatment with empagliflozin attenuates renal damage in obese zucker rat. Exp Clin Endocrinol Diabetes.

[4] Casazza K, Hanks LJ, Beasley TM (2011). Beyond thriftiness: independent and interactive effects of genetic and dietary factors on variations in fat deposition and distribution across populations. Am J Phys Anthropol.

[5] da Rocha FJ, Ogurtsova K, Linnenkamp U (2016). IDF Diabetes Atlas estimates of 2014 global health expenditures on diabetes. Diabetes Res Clin Pract.

[6] Khalili L, Alipour B, Asghari Jafarabadi M (2019). Probiotic assisted weight management as a main factor for glycemic control in patients with type 2 diabetes: a randomized controlled trial. Diabetol Metab Syndr.

[7] Evans G, Wright D. Long-term evaluation of a UK community pharmacy-based weight management service. Pharmacy 2020;8. 10.3390/pharmacy8010022. 10.3390/pharmacy8010022 PMC7151702 32092850

[8] Yandrapalli S, Jolly G, Horblitt A (2020). Cardiovascular safety and benefits of non-insulin antihyperglycemic drugs for the treatment of type 2 diabetes mellitus—part 1. Cardiol Rev.

[9] Forslund K, Hildebrand F, Nielsen T (2015). Disentangling type 2 diabetes and metformin treatment signatures in the human gut microbiota. Nature.

[10] Barengolts E (2016). Gut microbiota, prebiotics, probiotics, and synbiotics in management of obesity and prediabetes: review of randomized controlled trials. Endocr Pract Off J Am Coll Endocrinol Am Assoc Clin Endocrinol.

[11] Barengolts E, Smith ED, Reutrakul S (2019). The effect of probiotic yogurt on glycemic control in type 2 diabetes or obesity: a meta-analysis of nine randomized controlled trials. Nutrients.

[12] Atabati H, Esmaeili SA, Saburi E (2020). Probiotics with ameliorating effects on the severity of skin inflammation in psoriasis: evidence from experimental and clinical studies. J Cell Physiol.

[13] Yun SI, Park HO, Kang JH (2009). Effect of Lactobacillus gasseri BNR17 on blood glucose levels and body weight in a mouse model of type 2 diabetes. J Appl Microbiol.

[14] Rad AH, Abbasalizadeh S, Vazifekhah S (2017). The future of diabetes management by healthy probiotic microorganisms. Curr Diabetes Rev.

[15] Bayat A, Azizi-Soleiman F, Heidari-Beni M (2016). Effect of Cucurbita ficifolia and probiotic yogurt consumption on blood glucose, lipid profile, and inflammatory marker in type 2 diabetes. Int J Prev Med.

[16] Jalali SZ, Shiri MR, Shirazi MG (2020). Effect of probiotics on full intestinal feeding in premature infants: a double blind, Clinical Trial. Iran J Pediatr.

[17] Tonucci LB, Olbrich Dos Santos KM, Licursi de Oliveira L (2017). Clinical application of probiotics in type 2 diabetes mellitus: a randomized, double-blind, placebo-controlled study. Clin Nutr.

[18] Aliashrafi M, Nasehi M, Zarrindast M-R (2020). Association of microbiota-derived propionic acid and Alzheimer’s disease; bioinformatics analysis. J Diabetes Metab Disord.

[19] Kobyliak N, Conte C, Cammarota G (2016). Probiotics in prevention and treatment of obesity: a critical view. Nutr Metab.

[20] Howe K, Clark MD, Torroja CF (2013). The zebrafish reference genome sequence and its relationship to the human genome. Nature.

[21] Rendueles O, Ferrieres L, Fretaud M (2012). A new zebrafish model of oro-intestinal pathogen colonization reveals a key role for adhesion in protection by probiotic bacteria. PLoS Pathog.

[22] Shan Y, Fang C, Cheng C (2015). Immersion infection of germ-free zebrafish with Listeria monocytogenes induces transient expression of innate immune response genes. Front Microbiol.

[23] Hedrera MI, Galdames JA, Jimenez-Reyes MF (2013). Soybean meal induces intestinal inflammation in zebrafish larvae. PLoS ONE.

[24] Khoshnevisan K, Baharifar H, Torabi F (2021). Serotonin level as a potent diabetes biomarker based on electrochemical sensing: a new approach in a zebra fish model. Anal Bioanal Chem.

[25] Chan JK (2014). The wonderful colors of the hematoxylin**–**eosin stain in diagnostic surgical pathology. Int J Surg Pathol.

[26] Wittekind D (2003). Traditional staining for routine diagnostic pathology including the role of tannic acid. 1. Value and limitations of the hematoxylin–eosin stain. Biotech Histochem Off Publ Biol Stain Comm.

[27] Livak KJ, Schmittgen TD (2001). Analysis of relative gene expression data using real-time quantitative PCR and the 2(−Delta Delta C(T)) Method. Methods.

[28] Zang L, Maddison LA, Chen W (2018). Zebrafish as a model for obesity and diabetes. Front Cell Develop Biol.

[29] Goldsmith JR, Jobin C (2012). Think small: zebrafish as a model system of human pathology. J Biomed Biotechnol.

[30] Valcarce DG, Riesco MF, Martinez-Vazquez JM (2019). Diet supplemented with antioxidant and anti-inflammatory probiotics improves sperm quality after only one spermatogenic cycle in zebrafish model. Nutrients.

[31] Capiotti KM, Antonioli R, Kist LW (2014). Persistent impaired glucose metabolism in a zebrafish hyperglycemia model. Comp Biochem Physiol B Biochem Mol Biol.

[32] Gleeson M, Connaughton V, Arneson LS (2007). Induction of hyperglycaemia in zebrafish (Danio rerio) leads to morphological changes in the retina. Acta Diabetol.

[33] Faillaci F, Milosa F (2018). Obese zebrafish: a small fish for a major human health condition.

[34] Falcinelli S, Rodiles A, Hatef A (2017). Dietary lipid content reorganizes gut microbiota and probiotic *L. rhamnosus* attenuates obesity and enhances catabolic hormonal milieu in zebrafish. Sci Rep.

[35] Jensen DM, Hendricks KV, Mason AT (2020). Good Cop, bad cop: the opposing effects of macrophage activation state on maintaining or damaging functional β-cell mass. Metabolites.

[36] Flynn EJ, Trent CM, Rawls JF (2009). Ontogeny and nutritional control of adipogenesis in zebrafish (Danio rerio). J Lipid Res.

[37] Subbotin VM (2007). Analysis of arterial intimal hyperplasia: review and hypothesis. Theor Biol Med Model.

[38] Winer DA, Luck H, Tsai S (2016). The intestinal immune system in obesity and insulin resistance. Cell Metab.

[39] Hernandez-Santana YE, Giannoudaki E, Leon G (2019). Current perspectives on the interleukin-1 family as targets for inflammatory disease. Eur J Immunol.

[40] Yao K, Zeng L, He Q (2017). Effect of probiotics on glucose and lipid metabolism in type 2 diabetes mellitus: a meta-analysis of 12 randomized controlled trials. Med Sci Monit Int Med J Exp Clin Res.

[41] Kobyliak N, Falalyeyeva T, Mykhalchyshyn G (2018). Effect of alive probiotic on insulin resistance in type 2 diabetes patients: randomized clinical trial. Diabetes Metab Syndr.

[42] Zeinali F, Aghaei Zarch SM, Vahidi Mehrjardi MY (2020). Effects of synbiotic supplementation on gut microbiome, serum level of TNF-alpha, and expression of microRNA-126 and microRNA-146a in patients with type 2 diabetes mellitus: study protocol for a double-blind controlled randomized clinical trial. Trials.

[43] Yi CC, Liu CH, Chuang KP (2019). A potential probiotic Chromobacterium aquaticum with bacteriocin-like activity enhances the expression of indicator genes associated with nutrient metabolism, growth performance and innate immunity against pathogen infections in zebrafish (Danio rerio). Fish Shellfish Immunol.

[44] Azad MAK, Sarker M, Wan D (2018). Immunomodulatory effects of probiotics on cytokine profiles. Biomed Res Int.

[45] Delgadillo-Silva LF, Tsakmaki A, Akhtar N et al. Modelling pancreatic beta-cell inflammation in zebrafish identifies the natural product wedelolactone for human islet protection. Dis Models Mech. 2019; 12. 10.1242/dmm.036004 10.1242/dmm.036004 PMC6361155 30679186

[46] Tsarouchas TM, Wehner D, Cavone L (2018). Dynamic control of proinflammatory cytokines Il-1beta and Tnf-alpha by macrophages in zebrafish spinal cord regeneration. Nat Commun.

[47] Wang G, Liang R, Liu T (2019). Opposing effects of IL-1beta/COX-2/PGE2 pathway loop on islets in type 2 diabetes mellitus. Endocr J.

[48] Nguyen T, Payan B, Zambrano A (2019). Epigallocatechin-3-gallate suppresses neutrophil migration speed in a transgenic zebrafish model accompanied by reduced inflammatory mediators. J Inflamm Res.

