# Supplementary material for: Gut micobiota alteration by Lactobacillus rhamnosus reduces pro-inflammatory cytokines and glucose level in the adult model of Zebrafish
Source: BMC Res Notes. 2021 Aug 9;14:302. doi: 10.1186/s13104-021-05706-5 (PMC8351095; doi:10.1186/s13104-021-05706-5)
Supplement: Supplementary file 1 — Additional file 1. Complementary information of animals, probiotic administration, histological staining and quantitative real-time polymerase chain reaction analysis. [file 13104_2021_5706_MOESM1_ESM.docx]

**Additional file 1**

***Animals***

Adult *Danio rerio* AB strain was primarily received as a gift from Zebrafish core facility, University of Tampere, Finland. Fish were acclimated and subjected to cycles of breeding onward, under standard re-circulating sump tank conditions for further applications at Zebrafish Core Facility, Endocrinology and Metabolism Research Institute (EMRI), Tehran University of Medical Sciences, Tehran, Iran. Fish were kept in aerated water with a pH around 7.5, oxygen content around 6.5 ± 0.5 mg/L, water temperature around 28 ± 1°C and hardness of 250 mg/L CaCO3. Fish adjusted to a 14:10 hour light:dark cycle and water were replaced every other day [23]. The procedures used in this study adhered to the tenets of the Declaration of Helsinki. Random designated adult-bred males were transferred to specific incubators five days prior to experiments for acclimation. Fish fed with 20 mg/fish of commercial food containing 48% protein, 8% fat, and 2% fiber and freshly hatched Artemia every morning after turning on the lights. In this trial, 3 months old adult male fish were divided into four groups (two control group and two T2DM diabetic group) each one contained 15 fish. The experiments were performed in duplicate manner and duplicate grouping system. Each group was kept in 2-liter tanks under optimal conditions. The first healthy control group received no probiotic treatment (HC), the second healthy control group treated with probiotic (HC-P), the first T2DM group received no probiotic supplement (T2DM) and the second T2DM group received probiotic supplements (T2DM-P). Diabetic condition was induced in both T2DM groups by gradient hyper-glucose accumulation methodology, starting from 50 mM glucose concentration. Three days later, the glucose concentration raised to 100 mM, and at the end of the week it was increased to 200 mM. This gradual increasing of the glucose prevents fish fatality during conditioning period [24]. For biometric analysis*,* three fishes were randomly chosen from each group and allowed to reach a steady state( To avoid the stress effect) within few minutes in a separate beaker and euthanized by Eugenol (Sigma-Aldrich, St. Louis, MO) using 3 drops in 50 ml of RO water. Biometric parameters including fish length (from the head to the end of tail) and weight were measured and recorded and immediate blood glucose measurement was performed. Furthermore, body mass index (BMI) was measured based on dividing body weight (g) to the square of the body length (cm).

***Probiotic administration***

*Lactbacillus rhamnosus* GG (ATCC: 53103) capsule was purchased from Culturelle Probiotics Co., Canada. To reach the adequate amount of probiotic consumption, final concentration of 10^6^ colony-forming units (CFU)/ml of the bacteria was dissolved in RO water, and used for treatments designated for HC-P and T2DM-P groups. The water in HC-P and T2DM-P tanks were gently replaced by fresh probiotic-rich water and used for incubation and further experimental steps.

***Blood glucose measurements***

To collect the blood, tail posterior was cut toward the anus with sterile seizure along with light pressure on tail, one drop of blood directly applied to strip on a commercial glucometer in duplicate manner, Match™ (OK Biotech Co, Taiwan). Results were recorded and fish were then dissected to collect and preserve the intestinal tissue for histological analysis.

***Histological staining***

After collecting biometric results the same fish used for collecting intestinal sample. Intact intestine was removed and immediately stored in 10% formalin at +4°C. Following paraffin embedding, 10 µm sections were prepared from the mid- intestine (small intestine) and applied in hematoxylin and eosin (H&E) as well as Albert’s staining procedures [25,26] .

***Quantitative real-time polymerase chain reaction analysis***

To detect gene expression, total RNA was extracted from homogenized small intestine tissue using TRIzol reagent (Invitrogen, Carlsbad, CA). Reverse transcription was carried out with Gene Amp RNA PCR. Quantitative PCR (Applied Biosystems, Foster City, CA) was performed for each gene by a FluoCycle II TM Sybr Green master mix PCR kit using a standardized program (5' initial denaturing step at 95°C; 40 cycles of 15'' at 95°C, 20'' at 55°C, and 30'' at 72°C; melting point analysis in 0.1°C steps; final cooling step). All data were normalized to the expression of beta actin as housekeeping internal control gene. The primer sequences of IL-1β and TNF-α used for real-time PCR are summarized in table S1 (Supplementary Material). Relative quantification of target gene relative expression levels were calculated using ΔΔCt method [27].
