# Supplementary material for: Gut micobiota alteration by Lactobacillus rhamnosus reduces pro-inflammatory cytokines and glucose level in the adult model of Zebrafish
Source: BMC Res Notes. 2021 Aug 9;14:302. doi: 10.1186/s13104-021-05706-5 (PMC8351095; doi:10.1186/s13104-021-05706-5)
Supplement: Supplementary file 2 — Additional file 2: Table S1. Primer sequences used for amplification of specific genes through real-time quantitative PCR. [file 13104_2021_5706_MOESM2_ESM.docx]

**Table S1** Primer sequences used for amplification of specific genes through real-time quantitative PCR.

| Gene | Primer Sequences (5’ - 3’) | Product length(bp) | | Accession No | |
| --- | --- | --- | --- | --- | --- |
| IL-1β | Forward: CATTTGCAGGCCGTCACA  Reverse: GGACATGCTGAAGCGCACTT | 63 | NM_212844.2 | |  |
| TNF-α | Forward: CTGGATCTTCAAAGTCGGGTGTA  Reverse: TGTGAGTCTCAGCACACTTCCATC | 138 | AY_427649 | |  |
| β-Actin | Forward: ACAGGGAAAAGATGACACAGATCA  Reverse: CAGCCTGGATGGCAACGTA | 72 | NM_181601.5 | |  |

bp Base pair
