# Supplementary material for: Gut micobiota alteration by Lactobacillus rhamnosus reduces pro-inflammatory cytokines and glucose level in the adult model of Zebrafish
Source: BMC Res Notes. 2021 Aug 9;14:302. doi: 10.1186/s13104-021-05706-5 (PMC8351095; doi:10.1186/s13104-021-05706-5)
Supplement: Supplementary file 3 — Additional file 3: Table S2. Summary of the effects of probiotic supplementation on length, weight gain and BMI of experimental groups. [file 13104_2021_5706_MOESM3_ESM.docx]

**Table S2** Summary of the effects of probiotic supplementation on length, weight gain and BMI of experimental groups

| Group |  | | Length (cm) | | Weight (g) | | BMI (g/cm^2^) | |  | |  |
| --- | --- | --- | --- | --- | --- | --- | --- | --- | --- | --- | --- |
|  | Mean+ SE | | 36.733+ 0.328 ^b, c, d^ | | 0.496+ 0.006 ^b, c^ | | 0.368+ 0.011 ^b, c, d^ | |  | |  |
| HC | Range | | 36.10-37.20 | | 0.49-0.51 | | 0.35-0.39 | |  | |  |
|  | Mean+ SE | | 38.567+ 0.218 ^a, c, d^ | | 0.623+ 0.003^a, c, d^ | | 0.419+ 0.002^a,c, d^ | |  | |  |
| HC-P | Range | | 38.30-39.00 | | 0.62-0.63 | | 0.41-0.42 | |  | |  |
|  | Mean+ SE | | 34.866+ 0.088^a, b, d^ | | 0.343+ 0.008^a, b, d^ | | 0.282+ 0.008^a,b, d^ | |  | |  |
| T2DM | Range | | 34.70-35.00 | | 0.33-0.36 | | 0. 27-0.30 | |  | |  |
|  | | Mean+ SE | | 31.533+ 0.290^a, b, c^ | | 0.486+ 0.003 ^b, c^ | | 0.489 + 0.006^a,b, c^ | |  | |
| T2DM-P | | Range | | 31.00-32.00 | | 0.48-0.49 | | 0.48-0.50 | |  | |

a Significant difference of other groups vs. HC

b Significant difference of other groups vs. HC-P

c Significant difference of other groups vs. T2DM

d Significant difference of other groups vs. T2DM-P

The difference considered significant at p < 0.001, each group contained 6 zebrafish
